# Identification and expression analysis of Th2 immune-related gene IL4/13A in turbot (*Scophthalmus maximus*)

**DOI:** 10.3389/fimmu.2024.1500840

**Published:** 2024-12-03

**Authors:** Huanhuan Huo, Baoliang Liu, Zirui Wang, Qiubai Zhou

**Affiliations:** ^1^ College of Animal Science and Technology, Jiangxi Agricultural University, Nanchang, China; ^2^ Key Laboratory of Sustainable Development of Marine Fisheries of Ministry of Agriculture, Yellow Sea Fisheries Research Institute, Chinese Academy of Fishery Sciences, Qingdao, China; ^3^ Laboratory for Marine Fisheries Science and Food Production Processes, Qingdao National Laboratory for Marine Science and Technology, Qingdao, China

**Keywords:** *Scophthalmus maximus*, IL4/13A, Th2 immunity, immune response, gene identification

## Abstract

Th2 immunity is a primary host defense against extracellular pathogens, and different IL4/13 paralogues are involved in this immune response in fish. Here, we identified IL4/13A for further Th2 immune response providing information in turbot. The results showed that the full length of the IL4/13A gene is 1,333 bp, containing a 432-bp open reading frame (ORF) that encoded 144 amino acids. Phylogenetic analysis recently showed that turbot IL4/13A has a relationship with *Dicentrarchus labrax*. Moreover, syntenic analysis revealed similar neighboring genes associated with turbot IL4/13A, compared with other teleosts and mammals. In addition, IL4/13A was widely expressed in all examined tissues with the highest expression level in skin, followed by liver and gill. Finally, IL4/13A showed a general trend of upregulation in immune tissues following bacterial challenge. The significant quick induction of IL4/13A indicated its key roles to prevent pathogens. Characterizations of IL4/13A will probably contribute to understanding of Th2 immunity in fish.

## 1 Introduction

Interleukin-4 (IL-4) and interleukin-13 (IL-13) are Th2-type cytokines that drive canonical Th2 immunity in mammals (1). Although IL-4 and IL-13 have low amino acid sequence identity (<30%), they are really close. They are often activated together and participate in the same immune response (2–4). IL-4 and IL-13 are involved in adaptive immune responses by multiple Th2-mediated humoral immune responses and promoting CD4+ Th-cell differentiation, including Ig production, Ig class switching, B-cell proliferation, and B-cell activation (1, 5, 6). They act as anti-inflammatory agents by inhibiting the production of TNF-α, IL-1β, and IL-6. In addition, they can also induce programmed macrophages to differentiate into alternately activated macrophages (4, 7), thereby improving acute inflammatory response and promoting wound healing, tissue repair, and angiogenesis (8).

In fish, IL-4/13A and IL-4/13B function similarly to IL-4 and IL-13 in mammals (9–14). However, the low homology of IL4/13A makes it difficult to obtain in different fish especially those without a genome sequence. IL4/13A has been identified, and it provides a basal level of immunity. It has been reported that bony fish IL-4/13A can promote the proliferation and differentiation of B cells *in vivo* (15). After injecting recombinant IL-4/13A into zebrafish, T cells were also activated (16). In addition, IL-4Rα has been shown to bind to IL-4/13A *in vitro*, thereby interfering with IL-4/13A-mediated B-cell proliferation (12). In conclusion, the induction of Th2 by IL-4/13 homologues has been confirmed.

The origin and evolution of the Th2 immune response may be that a single IL-4/13 gene is replicated in different lineages by tandem duplication or whole genome duplication (WGD) events. In the second round (2R) WGD bony spotted fish (*Lepisosteus oculatus*) genome, a single IL-4/13 gene was found between KIF3A and RAD50 (4). Bony fish have two genes in the IL-4/13 family, IL-4/13A (adjacent to RAD50) and IL-4/13B (adjacent to KIF3A), which are most likely the result of round 3 (3R) WGD (17). Recently, four IL-4/13 loci were identified in the genomes of rainbow trout and Atlantic salmon, and three active genes IL-4/13A, IL-4/13B1, and IL-4/13B2 were cloned (14). With this likelihood, IL-4/13B1 and IL-4/13B2 have originated from the salmonid 4R WGD event (18).

In order to better understand the Th2 immune responses of turbot, we first reported the identification, molecular characterization, and IL-4/13A genomic loci in the turbot genome. Phylogenetic analysis showed that the IL4/13A gene had relatively low homology among different fish. The transcriptional expression before and after bacterial injection was compared and analyzed.

## 2 Materials and methods

### 2.1 Fish and sampling

Juvenile turbot weighing 150 ± 10 g were taken from the farm of Shandong Oriental Ocean Sci-Tech Co., Ltd. (Shandong, China). The fish adapted to the test environment for 7 days using a flow-through system. The fish were randomly assigned to six tanks, and among them, three were vaccinated with attenuated *E. tarda*. The immune method was exactly as described in our previous study (19). The experimental fish were injected with 100 µl attenuated *Edwardsiella tarda* (approximately 1 × 10^6^ colony forming units), and the control fish received 100 µl of 0.9% sterile physiological saline. Three fish were randomly collected at 0 h, 6 h, 12 h, 24 h, and 48 h from each tank immediately after anesthetization with 0.05% tricaine methane sulfonate (MS-222, Sigma, St. Louis, MO, USA). Samples at 0 h were used as control. Samples were flash-frozen in liquid nitrogen and stored at −80°C for standby.

### 2.2 Total RNA extraction

RNA was extracted using TRIzol reagents (Invitrogen, Carlsbad, CA, USA) according to the manufacturer’s instructions. RNA degradation was detected by 1% agarose gel electrophoresis. The quality of RNA was measured using the NanoDrop 2000 spectrophotometer (Thermo, New York, MA, USA). The RNA was then reverse-transcribed into cDNA for real-time fluorescence quantitative PCR assay.

### 2.3 Cloning and analysis of turbot IL-4/13A gene

RACE-PCR was performed using the 5′ and 3′-Full RACE Kit (TaKaRa, China). Primers F1/F2 and R3/R4 (**Table 1**) for the RACE-PCR were designed according to turbot IL-4/13A gene sequences predicted from our transcriptome database (SRA accession no. SRP129900). PCR products were cloned into the pMD18-T simple vector (TaKaRa, China), and primers RV-M/M13-47 were used for sequencing. Primers F5/R5 were used to verify the integrity of the IL4/13A gene sequence.

**Table 1:** Information of primers.

Name	Sequence(5′–3′)	Length
IL-4-F1	CACATCTGCCAGACACTTGACC	22
IL-4-F2	TGTTGGGGCCATAATTGTTTCAG	23
IL-4-R3	CCTTCACATCGGCCACAAACAGA	23
IL-4-R4	GAGAGCAGCAGGACCATCAGCGT	23
RV-M	GAGCGGATAACAATTTCACACAGG	24
M13-47	CGCCAGGGTTTTCCCAGTCACGAC	24
IL-4-F5	ATGTAGCAGACGCGTAGGCA	20
IL-4-R5	GTGCAGGTATAACTTCTTAG	20
IL-4-F6	GTCACCAAAGGTCCGGCTAA	20
IL-4-R6	GCAGATGTGTTCAGGCGTTG	20
β-Actin F	ATCGTGGGGCGCCCCAGGCACC	22
β-Actin R	CTCCTTAATGTCACGCACGATTTC	24

The sequence similarity analysis was carried out by the NCBI BLAST program. The Molecular Evolutionary Genetic Analysis (MEGA) software was used to construct the phylogenetic tree. Multiple-sequence alignment was performed by the Clustal X2 program. Signal peptide and glycosylation sites were predicted using Signal P4.0 software and the NetNGlyc1.0 server, respectively. The genomic structure of the IL-4/13A gene was analyzed by GeneWise software. The genes related to IL-4/13A in other vertebrate genomes were identified by Ensembl and GenBank databases, and the phylogenetic relationship between species was obtained.

### 2.4 Expression analysis of the turbot IL-4/13A gene

Reverse transcription was performed using the TaKaRa PrimeScript™ RT Reagent Kit with gDNA Eraser (Perfect Real Time). The gene-specific primer sets of F6/R6 (**Table 1**) were used for quantitative real-time PCR (qPCR). The β-actin gene was used as an internal parameter for quality control. β-Actin F and β-actin R as reference gene primers are provided in **Table 1**. The 25-μl reaction system was as follows: SYBR^®^ Premix Ex Taq п (Tli RNase H Plus (2×) 12.5 μl, upstream primer 1.0 μl (10 μM), downstream primer 1.0 μl (10 μM), cDNA template 2.0 μl (100 ng), and RNase Free dH_2_O 8.5 μl. Cycling conditions were as follows: 95°C for 1 min, followed by 40 cycles of 95°C 15 s, 60°C 15 s, 72°C 30 s. The fluorescence output of each cycle was measured and recorded for subsequent analysis. Each reaction had three parallels. Real-time quantitative PCR data analysis method: the 2^−△△Ct^ method was used in this experiment, and the calculation formula were as follows: (1) relative expression level (fold change) = 2^−△△Ct^; (2) △△Ct = (Ct target gene − Ct internal reference gene) treated group − (Ct target gene − Ct internal reference gene) untreated group.

### 2.5 Statistical analysis

The data were expressed as mean ± SD (standard deviation of the mean) and were analyzed in SPSS 19.0 software. Statistical significance was considered if *p* < 0.05.

### 2.6 Animal ethics

The experiment was performed in strict accordance with the guidelines and ethical principles of the Experiment Animal Welfare Ethics Committee of China. The experimental design was approved by the Committee on Research Ethics of the Department of Laboratory Animal Science, Jingxi Agricultural University. All efforts were made to minimize fish suffering. Approval Code: JAU-2020-0021.

## 3 Results and discussion

### 3.1 Identification and characterization of turbot IL-4/13A

**Figure 1:**
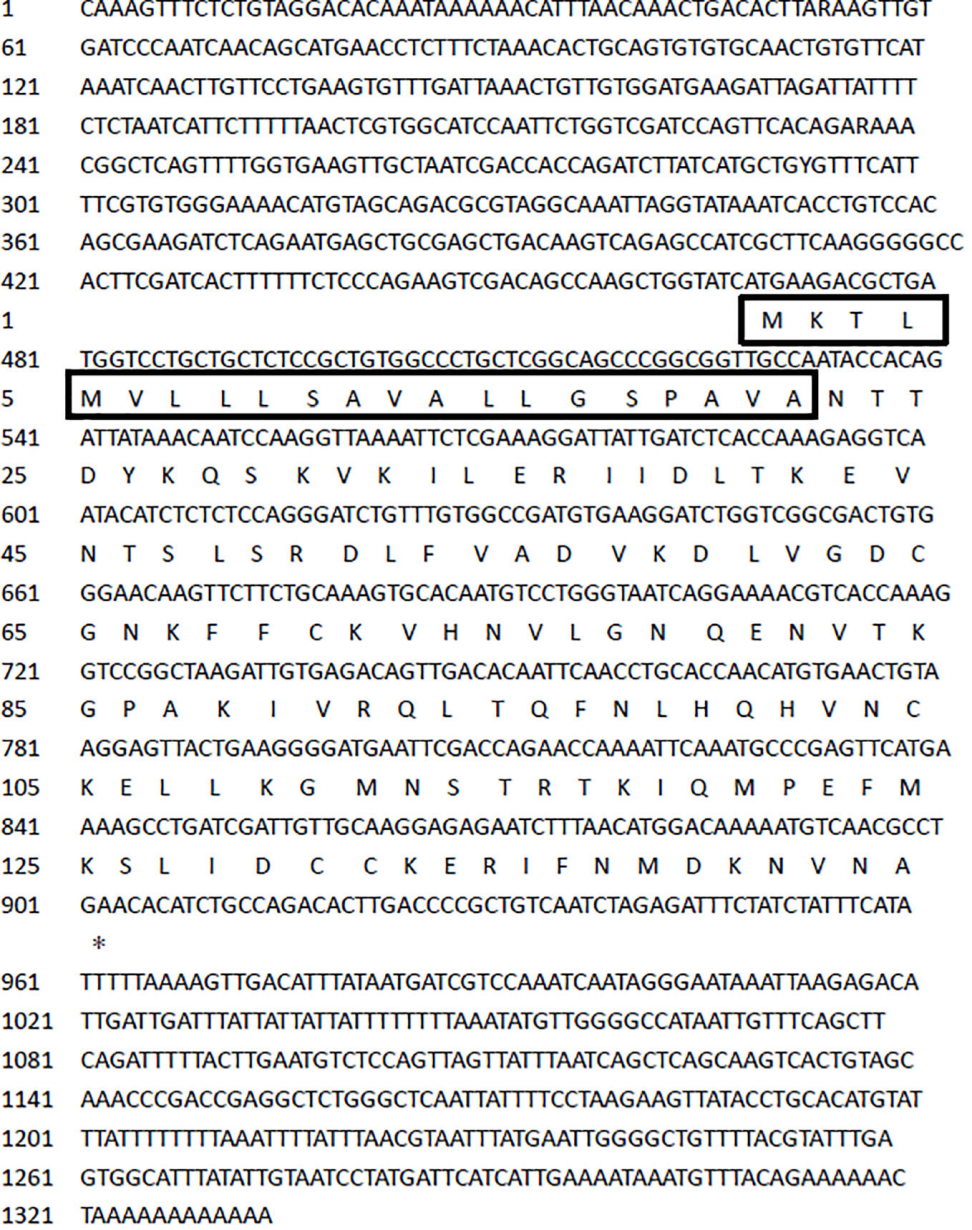
The determined cDNA sequence of the IL-4/13 gene is 1,333 bp, encoding 144 amino acids. The black box denotes predicted signal peptides of 21 amino acids. * is termination codon.

The results have been submitted to NCBI (BankIt2748472 Seq OR621078). The complete cDNA sequence of IL-4/13 was 1,333 bp, containing a 432-bp open reading frame (ORF) that encoded 144 amino acids (**Figure 1**), with predicted signal peptides of 21 amino acids and 4 potential N-glycosylation sites (**Table 2**). The theoretical molecular weight of the turbot IL-4/13 mature peptide was 16.2 kDa. The turbot IL-4/13 has a basic pI 9.28 (**Table 2**). The instability index of turbot IL-4/13 is 21.06 belonging to stable protein. For all of fish IL-4/13A, the protein of turbot IL-4/13 is hydrophilic and the grand average of hydropathicity of turbot IL-4/13 is −0.130. As can be seen from **Table 2**, the characterizations of turbot IL-4/13 were similar with other fish IL-4/13A.

**Table 2:** Characterizations of turbot IL-4/13 and other fish IL-4/13A.

Features Fish	Full length (aa)	Signal peptide (aa)	Mature peptide	Cysteine residues	Disulfide bonds	pI^a^	MW^b^	N-Glycosylation sites	Instability index	GRAVY^c^
Turbot IL-4/13	144	21	123	5	2	9.28	16.20	4	21.06 stable	−0.130
Seabass IL-4/13A1	144	23	121	4	1	7.71	16.32	3	60.73 unstable	−0.118
Seabass IL-4/13A2	142	23	119	6	2	8.97	16.19	1	43.06 unstable	−0.335
Trout IL-4/13A	145	26	119	4	1	7.01	16.16	0	38.84 stable	−0.161
Salmon IL-4/13A	142	25	117	4	1	8.93	16.05	2	33.39 stable	−0.222
Grass carp IL-4/13A	135	15	120	4	2	8.33	15.62	0	30.09 stable	−0.382
Zebrafish IL-4/13A	157	16	142	5	2	7.62	17.81	0	39.17 stable	−0.429
Fugu IL-4/13A	140	25	115	5	2	8.88	15.74	4	18.95 stable	−0.025
Pufferfish IL-4/13A	139	19	120	5	2	8.63	16.13	4	40.08 unstable	−0.527
Crucian carp IL-4/13A	137	15	122	4	2	6.3	15.82	2	29.19 stable	−0.527

^a^pI of predicted mature peptides.

^b^Theoretical molecular weight (kDa) of the predicted mature peptides.

^c^Grand average of hydropathicity of the predicted mature peptides.

A BLAST search was performed to determine the homologous amino acids to other species. The turbot IL-4/13 shares low identity (32%–35%) to IL-4/13A from *Sparus aurata*, *Tetraodon nigroviridis*, and *Dicentrarchus labrax*. The IL-4/13A gene which has low homology was also found in salmonid, sea bass, yellow croaker, tetraodon, and so on (14, 20–22). The homology between tetrapods and tetraodon is <15%. Low homology demonstrated that IL-4/13 genes were fast evolving.

**Figure 2:**
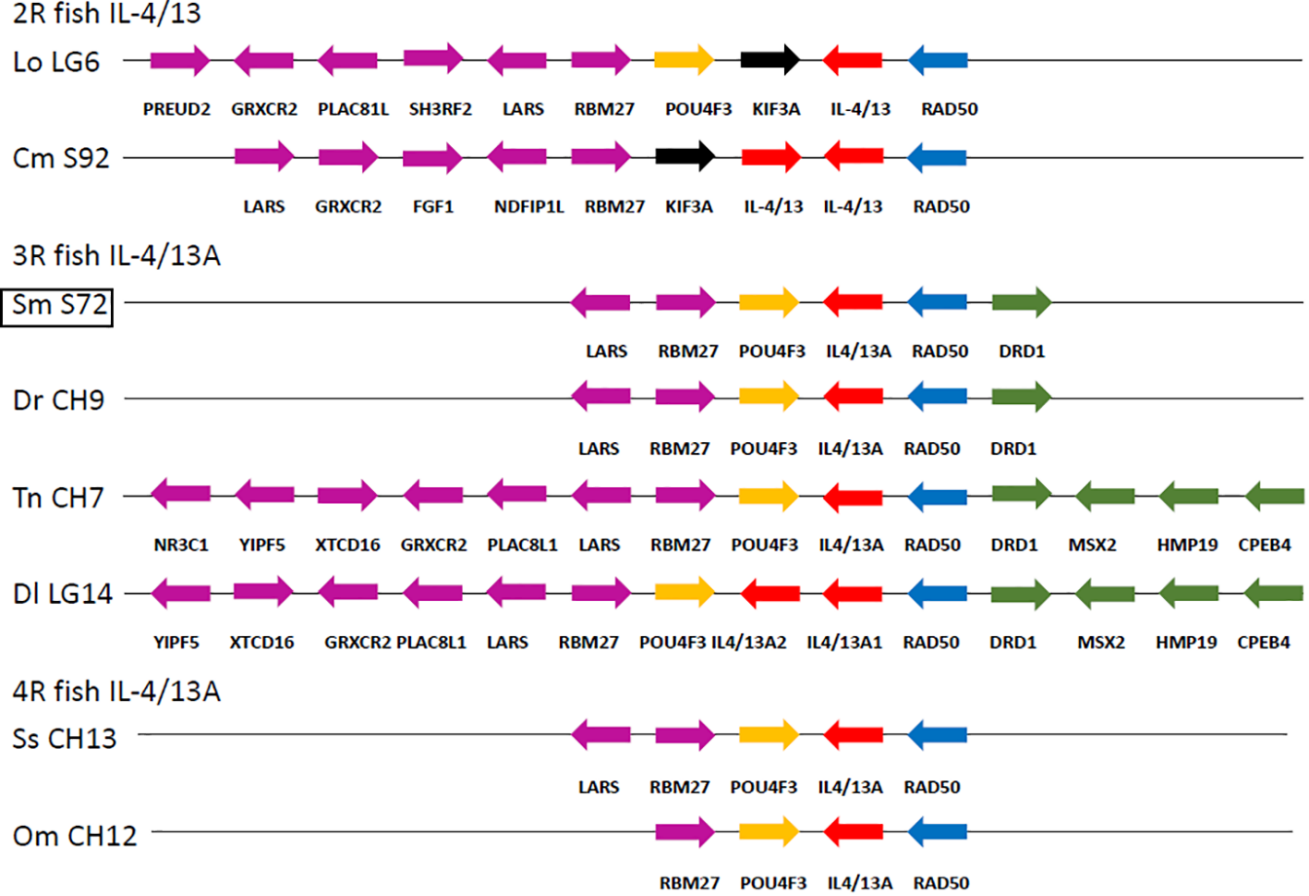
Gene synteny of IL-4/13A loci across bony fish. Evolutionarily relevant genes located at the 5′ and at the 3′ of the IL-4/13 loci are evidenced, and their strand orientations are indicated with arrows. The immediately flanking (POU4F3/RAD50) and neighboring genes at each side of the 2R/3R/4 fish IL-4/13 loci are differently colored using the European sea bass genomic organization as a reference. Lo, *Lepisosteus oculatus*; Cm, *Callorhinchus milii*; Sm, *Scophthalmus maximus*; Dr, *Danio rerio*; Tn, *Tetraodon nigroviridis*; Dl, *Dicentrarchus labrax*; Ss, *Salmo salar*; Om, *Oncorhynchus mykiss*.

In the IL-4/13A locus, we further identified a highly conserved homologous gene segment in bony fish (**Figure 2**). The turbot IL-4/13 gene was adjacent to the RAD50 gene, which was exactly the same as previously reported for other teleost IL-4/13A (23). There are at least six conserved genes (containing, LARS, RBM27, POU4F3, IL-4/13A, RAD50, and DRD1) that are identical with 3R teleost *Danio rerio* and *Tetraodon nigroviridis* (**Figure 2**). This suggested the turbot IL-4/13 is IL-4/13A.

### 3.2 Phylogenetic analyses

**Figure 3:**
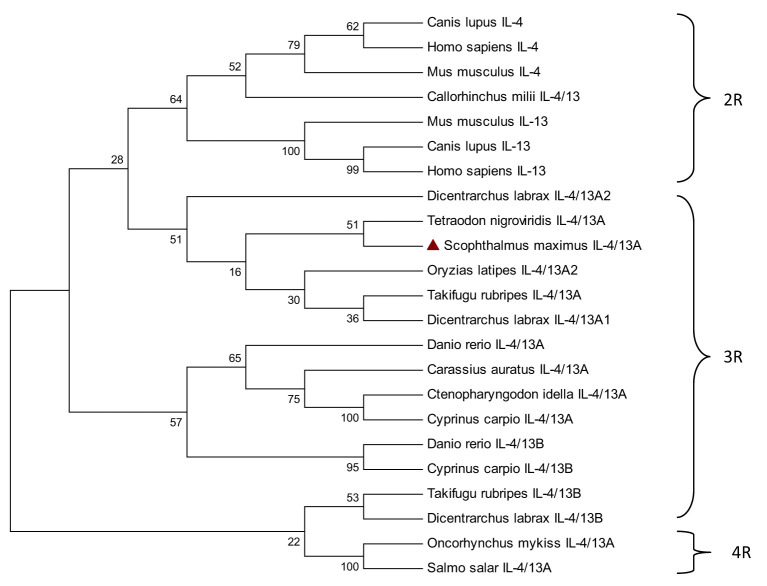
Phylogenetic tree analysis of fish IL-4/13 with mammalian IL-4/IL-13.

To gain a deeper understanding of the evolution of IL-4/13A, a phylogenetic tree was constructed based on multiple comparisons of IL-4/13A, IL-4/13B in fish, and IL-4 and IL-13 in mammals (**Figure 3**). The IL-4/13 of 2R fish *Callorhinchus milii* and IL-4 and IL-13 of 2R mammals formed a cluster with 64% bootstrap support. The turbot IL-4/13A, along with the IL-4/13A from some 3R fish (*Dicentrarchus labrax*, *Tetraodon nigroviridis*, *Oryzias latipes*, and *Takifugu rubripes*), formed a cluster with 51% bootstrap support. Then, the two formed a large cluster with 28% bootstrap support. Although the bootstrap support is low, they are more closely related compared with the IL-4/13A and IL-4/13B molecules from some other 3R fish (*Danio rerio*, *Carassius auratus*, *Ctenopharyngodon idella*, *Cyprinus carpio*). The IL-4/13A molecules form 4R fish (*Oncorhynchus mykiss* and *Salmo salar*) and the IL-4/13B molecules from 3R fish (*Takifugu rubripes* and *Dicentrarchus labrax*) are furthest with turbot IL-4/13A. These suggested that the differentiation of these IL-4/13 genes in fish occurs after the third round (3R) of whole genome duplication (WCG). This result agrees with Ohtani (13). Meanwhile, the IL-4/13 gene is still differentiated in 4R fish.

### 3.3 Structural features of turbot IL-4/13A

**Figure 4:**
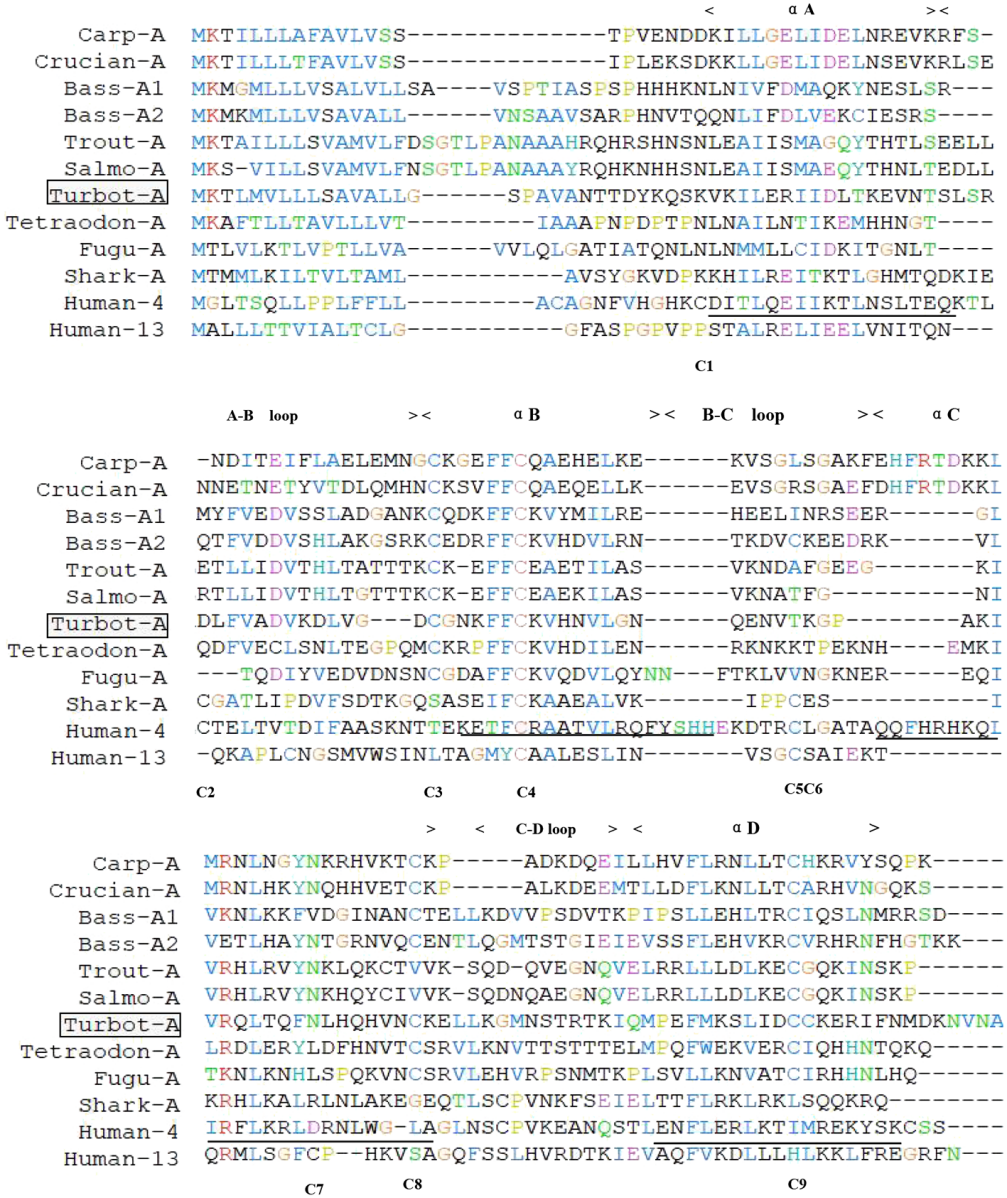
Amino acid sequence alignment of the predicted turbot IL4-13A with selected IL4-13 molecules. The position of the nine cysteine residues found in the different sequences is highlighted below the alignment and shown belong the sequences. Human-4 C5 and Huan-13 C6 are on the same vertical line. The four α-helices and loop regions known for human IL-4 are shown above the alignment and the amino acids involved in the α-helices underlined in the human IL-4 sequence.

In teleost IL-4/13A, four to six cysteine residues were found and the residues are in different positions. The nine cysteine residue positions conserved in different lineages (14) are highlighted below the sequences (**Figure 4**). The only conserved cysteine residue in all sequences was C4. Turbot IL-4/13A contains four conserved cysteine residues Cys^64^, Cys^70^, Cys^104^, and Cys^130^ (conserved positions corresponding to C3, C4, C8, and C9, respectively) and one additional cysteine residue Cys^131^; as predicted for 3R of fish IL-4/13A, they should form two disulfide bridges (C3 and C4, C8, and C9) (24). The predicted alpha helix of turbot IL-4/13A is 62.50%, the random coil is 36.11%, and the extended strand is 1.39%.

### 3.4 Expression analyses of turbot IL-4/13A

**Figure 5:**
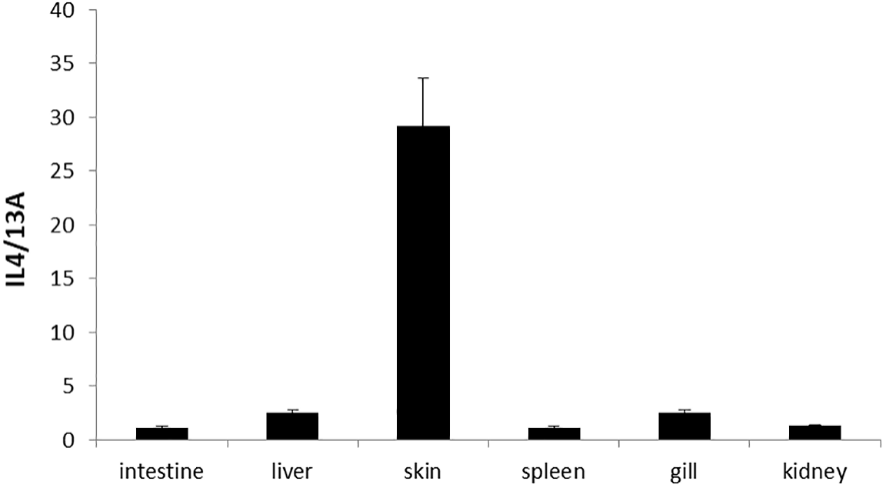
Tissue distribution of IL-4/13A in turbot. The data were expressed as mean ± SD.

The expression of turbot IL-4/13A could be detected in the kidney, spleen, intestine, gill, liver, and skin by real-time qPCR (**Figure 5**). In general, the expression level was highest in skin, followed by liver, gill, kidney, and spleen, and lowest in intestine (**Figure 3**). The expression level of IL-4/13A was similar to large yellow croaker (*Larimichthys crocea*) and Atlantic salmon (*Salmo salar*) (21, 25). Interesting to note is that the lowest expression of IL-4/13A1 was found in gill in sea bass (*Dicentrarchus labrax*) (17). The expression of IL-4/13A2 was abundant in gill in sea bass. The IL-4/13A gene was further differentiated into two genes in sea bass. The regulation of type 2 immune response in fish is complicated due to the existence of multiple IL-4/13 subtypes.

**Figure 6:**
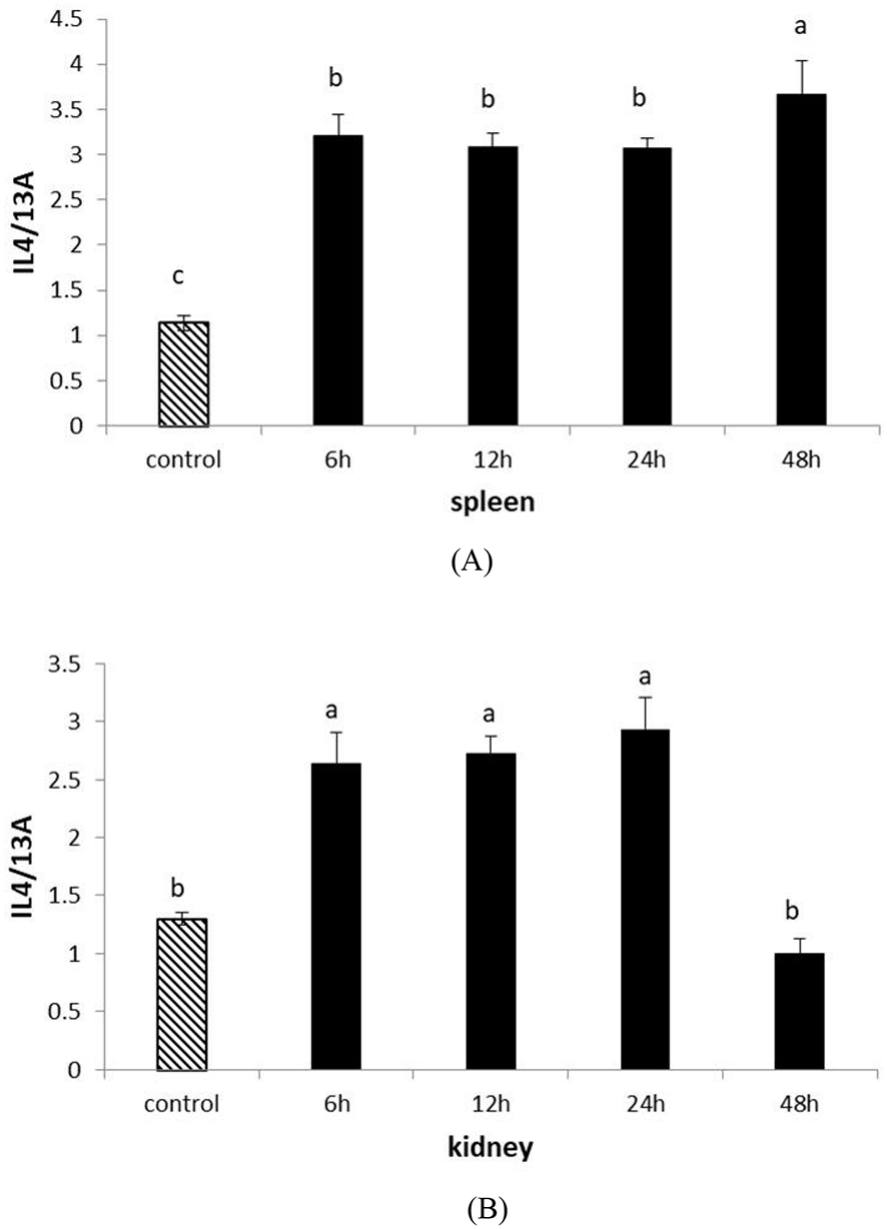
The expression of turbot IL-4/13A after stimulation with attenuated *Edwardsiella tarda* in kidney **(A)** and spleen **(B)**. The data were expressed as mean ± SD. The relative significance of an LSD *post-hoc* test after a significant one way-ANOVA between different time points. Different lowercase letters represent significant differences (*p < 0.05*).

To further understand the role of IL-4/13A in the immune response of turbot, we examined its expression after inoculation with attenuated *Edwardsiella tarda* vaccine. As we focused on immunity, the spleen and kidney were selected. Following attenuated *Edwardsiella tarda* challenge, IL-4/13A expression increased rapidly in both spleen and kidney. From 6 h to 24 h postinfection, IL-4/13A expression was downregulated in spleen (**Figure 6A**). IL-4/13A expression touched the peak at 48 h postinfection by 3.2-fold relative to the control fish in spleen. In the kidney, IL-4/13A expression was upregulated gradually and touched the peak at 24 h by 2.2-fold, and then quickly plummeted to a minimum at 48 h (**Figure 6B**). Similar results were found in salmon (25) and large yellow croaker (21) after vaccine infection.

In conclusion, we identified the IL-4/13A gene from turbot and analyzed its characters and expression pattern. Turbot IL-4/13A is broadly and highly expressed and has potent bioactivities at low concentrations, which may provide a basal level of type 2 immunity. Further studies are needed to identify and analyze the homologous genes, gaining a deeper understanding of the evolution of type 2 immunity in turbot.

## Data Availability

The experiment was performed in strict accordance with the guidelines and ethical principles of the Experiment Animal Welfare Ethics Committee of China. The experimental design was approved by the Committee on Research Ethics of the Department of Laboratory Animal Science, Jingxi Agricultural University. All efforts were made to minimize fish suffering. Approval Code: JAU-2020-0021.
